# Nuclear FABP7 regulates cell proliferation of wild‐type *IDH1* glioma through caveolae formation

**DOI:** 10.1002/1878-0261.13130

**Published:** 2021-11-09

**Authors:** Yoshiteru Kagawa, Banlanjo Abdulaziz Umaru, Masayuki Kanamori, Ryo Zama, Subrata Kumar Shil, Hirofumi Miyazaki, Shuhei Kobayashi, Tunyanat Wannakul, Shuhan Yang, Teiji Tominaga, Yuji Owada

**Affiliations:** ^1^ Department of Organ Anatomy Tohoku University Graduate School of Medicine Sendai Japan; ^2^ Department of Neurosurgery Tohoku University Graduate School of Medicine Sendai Japan

**Keywords:** acetyl‐CoA, caveolae, caveolin‐1, fatty acid‐binding protein 7, isocitrate dehydrogenase 1

## Abstract

Isocitrate dehydrogenase 1 (IDH1) is a key enzyme in cellular metabolism. *IDH1* mutation (IDH1mut) is the most important genetic alteration in lower grade glioma, whereas glioblastoma (GB), the most common malignant brain tumor, often has wild‐type *IDH1* (IDH1wt). Although there is no effective treatment yet for neither IDH1wt nor IDHmut GB, it is important to note that the survival span of IDH1wt GB patients is significantly shorter than those with IDH1mut GB. Thus, understanding IDH1wt GB biology and developing effective molecular‐targeted therapies is of paramount importance. Fatty acid‐binding protein 7 (FABP7) is highly expressed in GB, and its expression level is negatively correlated with survival in malignant glioma patients; however, the underlying mechanisms of FABP7 involvement in tumor proliferation are still unknown. In this study, we demonstrate that FABP7 is highly expressed and localized in nuclei in IDH1wt glioma. Wild‐type *FABP7* (FABP7wt) overexpression in IDH1wt U87 cells increased cell proliferation rate, caveolin‐1 expression, and caveolae/caveosome formation. In addition, FABP7wt overexpression increased the levels of H3K27ac on the caveolin‐1 promoter through controlling the nuclear acetyl‐CoA level via the interaction with ACLY. Consistent results were obtained using a xenograft model transplanted with U87 cells overexpressing *FABP7*. Interestingly, in U87 cells with mutant *FABP7* overexpression, both *in vitro* and *in vivo* phenotypes shown by FABP7wt overexpression were disrupted. Furthermore, IDH1wt patient GB showed upregulated caveolin‐1 expression, increased levels of histone acetylation, and increased levels of acetyl‐CoA compared with IDH1mut patient GB. Taken together, these data suggest that nuclear FABP7 is involved in cell proliferation of GB through caveolae function/formation regulated via epigenetic regulation of caveolin‐1, and this mechanism is critically important for IDH1wt tumor biology.

## Graphical abstract

In this study, we observed high expression and nuclear localization of FABP7 in IDH1wt glioblastoma (GB). Using GB cell lines, we investigated that nuclear FABP7 interacted with ATP citrate lyase (ACYL), as well as the associated increase of nuclear acetyl‐CoA levels, which led to an increase of histone acetylation at the caveolin‐1 promoter. Consequently, the increase in caveolin‐1 expression enhanced caveolae/caveosome function and formation, as well as the intracellular signalling activity in IDHwt cells related to GB tumour proliferation.

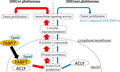

**Abbreviations**
ACLY: ATP citrate lyase
D2HG: D‐2 hydroxyglutarate
DAPI: diamidino‐2‐phenylindole
DHA: docosahexaenoic acid
DIV: day *in vitro*
FABP7: fatty acid‐binding protein 7
FAs: fatty acids
FBS: fetal bovine serum
GB: glioblastoma
IDH: isocitrate dehydrogenase
qPCR: quantitative real‐time PCR
WHO: World Health Organization
αKG: α‐ketoglutarate

## 1 Introduction

Gliomas, the most common and deadly brain tumors, arise from glial or glial progenitor cells in the central nervous system. These tumors have been classified by the World Health Organization (WHO) into three malignancy grades: II, III, and IV based on morphology and cell type [1]. Glioblastoma (GB) classified as grade IV is the most malignant brain tumor particularly due to its rapid proliferative, infiltrative, and invasive biological characteristic [2, 3]. Additionally, isocitrate dehydrogenase (IDH) mutations have been employed as significant prognostic biomarkers as there is documented favorable outcomes for patients with mutated IDH (IDHmut) with their survival span longer than glioma patients with wild‐type IDH (IDHwt) [1, 4]. Despite the considerable therapeutic progress achieved in neurosurgery, radiotherapy, and chemotherapy, GBM remains an incurable disease because of its radio‐resistance and the difficulty of drug delivery across blood–brain barrier. Consequentially, the median survival time of GBM patients with IDHwt is 1.1 and 3.7 year for GBM patients with IDHmut [5]. Thus, a comprehensive characterization of GBM biology, especially IDHwt, and exploring other effective molecular targeted therapies that could improve the diagnostic accuracy and individualized treatments is of utmost importance.

Fatty acid‐binding proteins (FABPs) are intracellular fatty acids (FAs) chaperon that control the uptake, metabolism, and storage of FAs. Among 11 subtypes of FABPs, FABP7 is drastically expressed in GB following previous WHO classifications [6] and its expression level is correlated with malignancy and survival in malignant glioma patients [7, 8]. In addition, FABP7 is highly expressed in melanoma [9], breast cancers [10], renal cancers [11], regulating their proliferation, and its expression level in these tumors is correlated with patient survival. Given these correlations, FABP7 is thought to be one of the tumorigenesis markers, but the underlying mechanisms of FABP7 involvement in tumor proliferation are still unknown.

FABP7 is also found in astrocytes and oligodendrocyte precursor cells in physiological brain [12] and involved in astrocyte proliferation [12, 13]. Previously, we found that FABP7 in astrocytes controls the function of caveolae, which are the main source of cellular activity in response to external stimuli, via regulating the expression of caveolin‐1, a specific scaffold protein in caveolae [14]. Further mechanistic analysis revealed that nuclear FABP7 in astrocytes regulates nuclear acetyl‐CoA production via interacting with ATP citrate lyase (ACLY), followed by epigenetically controlling caveolin‐1 expression [15]. These results suggest that nuclear FABP7‐regulated caveolae function may influence cellular activity in physiological astrocytes and possibly in pathophysiological tumors. Of note, nuclear localization of FABP7 demonstrated by wild‐type FABP7 overexpression in U87 cells shows higher rate of cell migration compared with control cells, but cytoplasmic localization of FABP7 demonstrated by lipid binding domain mutation of FABP7 overexpression does not affect migration [16], indicating the importance of nuclear FABP7 in GB; however, detailed molecular mechanism of nuclear FABP7 is still unknown.

In this study, we have explored the expression and localization of FABP7 in current separated grade of glioma with focus on IDH1 mutation and how FABP7 regulates cellular activity of GB focusing on the epigenetic regulation of caveolin‐1.

## 2 Materials and methods

### 2.1 Patient sample

This study was complied with principles in the Declaration of Helsinki and conducted with the approval of the ethics committee of Tohoku University School of Medicine (2019‐1‐323). The experiments were undertaken with the understanding and written consent of each subject. A total of nine frozen samples (two are IDH1mut diffuse glioma, two are IDH1mut anaplastic glioma, three are IDH1mut GB, two are IDH1wt GB) were used for western blot experiments. A total of 15 paraffin‐embedded samples (each three samples of IDH1mut diffuse glioma, IDH1wt and IDH1mut anaplastic glioma, IDH1wt and IDH1mut GB) were used for immunohistological experiments. A total of 12 frozen samples (six were IDH1wt GB and six were IDH1mut GB) were used for acetyl‐CoA measurement.

### 2.2 TCGA database

Provisional datasets from TCGA and cBioPortal were used for the analysis to examine the expression level of FABP7 between IDH1wt and IDH1mut diffuse glioma (*n* = 506) and between each genotype of astrocytoma (*n* = 192), oligodendrocytoma (*n* = 131), and oligodendroglioma (*n* = 181). First, we categorized the expression level of FABP7 in diffuse glioma for Table 1 and astrocytoma/oligodendrocytoma/oligodendroglioma for Table 2 using cBioPortal database. Then, we checked the status of IDH1 mutation in each glioma sample using TCGA database.

### 2.3 Cells

U87 and U251 human GB cell line were obtained from ATCC and came with comprehensive authentication and quality controls. The cells were maintained by passage in Dulbecco’s modified Eagle’s medium (DMEM, Thermo Fisher Scientific Inc., Waltham, MA, USA) containing 10% (v/v) heat‐inactivated fetal bovine serum (FBS) (Thermo Fisher Scientific Inc.) and 1% (v/v) penicillin/streptomycin (Thermo Fisher Scientific Inc.).

To obtain FABP7 knock‐out (KO) cells in U251 cells, we performed Crispr/Cas9 editing as previously described [17]. Briefly, sgRNA expression plasmid was constructed using CHOPCHOP (https://chopchop.cbu.uib.no/), with selected target sites within exon 1 of the human FABP7 gene. The following oligonucleotides were used: gRNA1; Forward 5′‐CTCATCAAAGTTCTGACTGT‐3′ and Reverse 5′‐GGAAGCTGACCAACAGTCAG‐3′, gRNA2; Forward 5′‐GAACTTTGATGAGTACATGA‐3′ and Reverse 5′‐TCATGTACTCATCAAAGTTC‐3′. The synthesized double‐stranded oligonucleotides were then inserted into a pGuide‐it‐ZsGreen1 Vector (Takara, Tokyo, Japan) following the manufacturer’s instructions. Constructed vector was transfected into U251 cells using Lipofectamine® 2000 Reagent (Thermo Fisher Scientific Inc.). The culture medium was changed the next day and after 48 h of transfection, GFP‐positive cells were sorted as single clones using a flow cytometer FACS Aria II (BD Bioscience, Franklin Lakes, NJ, USA). After obtaining monoclonal cell lines, genomic DNA was extracted from cells and amplified using the following primer set: Forward 5′‐ACTCGACCTACTCCGCTAACC‐3′ and Reverse 5′‐CTCTTCTCCCAGCTGGAAACTAA‐3′ Sequencing analysis was performed to screen genomic mutation using the forward primer. Nonedited cells by gRNA1 treatment were used as U251 control cells.

Human FABP7 (FABP7wt) expression vector and it with point mutation (R109A/R129A) (FABP7mut) were generated previously [18]. Each fragment excised by restriction enzymes was inserted into pCW lentivirus vector. pCW‐vector or pCW‐FABP7wt or pCW‐FABP7mut was transfected into HEK293T cells with pCAG‐HIVgp and pCMV‐VSV‐G‐RSV‐Rev using lipofectamine 2000 (Thermo Fisher Scientific Inc.). Next day, the culture medium was replaced with fresh medium. Forty‐eight hours after the medium change, the viral supernatants were collected and filtered. U87 cells were suspended in the medium containing the virus. FABP7‐expressing cells, which were venus‐positive, were collected using a flow cytometer FACS Aria II (BD Bioscience). Each series of sorted cells was seeded and venus‐positive expression was confirmed by confocal scanning‐laser microscopy (Zeiss LSM780 META, Carl Zeiss, Oberkochen, Germany). To induce the expression of FABP7wt or FABP7mut, medium was replaced with fresh medium containing 5 μg·mL^−1^ doxycycline (Wako, Osaka, Japan).

To obtain stable expression of luciferase in U87 cells, the sequence of luciferase was carried in pCS2 vector with tdTomato sequence. pCS2‐luciferase‐IRES2‐tdTomato vector was transfected into HEK293T cells with pCAG‐HIVgp and pCMV‐VSV‐G‐RSV‐Rev using lipofectamine 2000. Luciferase‐expressing cells, which were tdTomato‐positive, were collected using a flow cytometer FACS Aria II and sorted cell was seeded in each plate and tdTomato‐positive expression was confirmed by confocal scanning‐laser microscopy. Then, FABP7wt or FABP7mut were expressed with the system above mentioned.

### 2.4 Immunocytochemistry and immunohistochemistry

For immunocytochemistry, culture dishes containing cells were washed with D‐PBS (‐) twice and fixed with 4% paraformaldehyde (PFA; Nacalai Tesque, Kyoto, Japan). Fixed cells were permeabilized with 0.3% (v/v) Triton X‐100 in PBS and blocked with FBS in PBS. The reaction with primary antibodies (as shown in Table S1) was performed overnight at 4 °C, and the reaction with secondary antibodies (as shown in Table S1) and DAPI was performed for 1 h at room temperature. Immunohistochemistry was performed as described previously [19]. Briefly, mice were transcardially perfused with 4% PFA in PBS, and paraffin sections (4 μm) were prepared from brain. Hematoxylin and eosin staining was performed using standard methods. For DAB (3,3′‐Diaminobenzidine) staining and immunofluorescence staining, sections were incubated with primary antibodies and the appropriate secondary antibodies. Visualization was performed using Vectastain ABC reagent (Vector Laboratories, Burlingame, CA, USA). For immunofluorescence staining, diamidino‐2‐phenylindole (DAPI, Thermo Fisher Scientific Inc.) is used for counterstaining. Observation was performed using confocal laser microscopy. For enzyme‐based immunohistochemistry, biotinylated rabbit anti‐goat IgG was used as a secondary antibody followed by reaction with 3,3′‐diaminobenzidine tetrahydrochloride (Sigma‐Aldrich, St. Louis, MO, USA) using VECTASTAIN Elite ABC kit (Vector Laboratories, Burlingame, CA, USA) under the manufacturer’s instructions. Samples were examined by microscopy (BX50, Olympus Corporation, Tokyo, Japan). For the quantification of the immune‐positive cells, we blindly and randomly obtained at least three images from the stained section of each genotype and immune‐positive cells in the image were counted and normalized with hematoxylin‐positive cells.

### 2.5 Proliferation assay

Proliferation was evaluated by counting using CellDrop Automated Cell Counters (DeNovix, Wilmington, DE, USA) and cell count reagent SF (Nacalai Tesque) following the manufacturer’s instructions.

### 2.6 Quantitative real‐time PCR

Total RNA was extracted using an RNeasy plus Mini kit (Qiagen, Hilden, Germany). Total RNA (4 µg) was reverse‐transcribed using anchored‐oligo(dt)18 primers (Transcriptor High Fidelity cDNA Synthesis Kit; Roche, Basel, Switzerland). Quantitative real‐time PCR (qPCR) was performed using SYBR® Premix Ex Taq™ II (Takara). Target gene primers are as follows: human CAV1, Fw5′‐GCGACCCTAAACACCTCAAC‐3′ and Rv5′‐ATGCCGTCAAAACTGTGTGTC‐3′, human GAPDH, Fw5′‐GGAGCGAGATCCCTCCAAAAT‐3′ and Rv5′‐GGCTGTTGTCATACTTCTCATGG‐3′. Relative gene expression was calculated using the $2‐\Delta \Delta C_{t}$ method.

### 2.7 Western blot

The cell lysates were resolved by SDS/PAGE and transferred to a polyvinylidene difluoride membrane (Merck Millipore, Burlington, MA, USA). The membrane was incubated with primary antibody (as shown in Table S1) overnight at 4 °C followed by incubation with secondary antibody (as shown in Table S1). Detection was performed with the ECL Detection Kit (Thermo Fisher Scientific Inc.).

### 2.8 Chromatin immunoprecipitation

Chromatin immunoprecipitation was performed as reported previously [15]. The relative levels of histone modifications of each target sequence were analyzed by qPCR using SYBR® Premix Ex Taq™ II (Takara) and Light Cycler 1.5® Carousel‐based system (Roche). The primers to amplify the several promoter regions for *Cav1* were as follows: Fw5′‐AAACGTTCTCACTCGCTCTCTGCT‐3′ and Rv5′‐GCGAGCAGAACAAACCTTG‐3′ for −273 bp to −148 bp from start codon as previously reported [20], Fw5′‐GCAGCACACGTCCGGGCCAACCGCG‐3′ and Rv5′‐ CCCACGGATGCCTACCTCCGAGTC‐3′ for −190 bp to +45 bp from start codon.

### 2.9 Electron microscopy analysis

Cultured U87 cells were fixed in 2% formaldehyde and 2.5% glutaraldehyde (JEOL, Tokyo, Japan) in PBS for 30 min. The samples were then washed with PBS and postfixed in 2% osmium tetroxide (JEOL) for 30 min. All samples were washed twice in PBS, dehydrated in ascending grades of ethanol and embedded in TAAB Epon 812 (TAAB, Reading, UK). Serial sections (70 nm in thickness) were cut on an Ultracut UCT ultramicrotome (Leica, Buffalo Grove, IL, USA). After being stained with uranyl acetate and lead citrate, they were examined on a JEM 1400 transmission electron microscope (JEOL) operated at 80 kV. Digital images were collected by a JEOL charge‐coupled device (CCD) camera (JEOL).

Caveolae are specialized invaginations of the plasma membrane found in numerous cell types and caveosome is defined as caveolae‐related endosome [21, 22]. As following previous reports, quantitative analysis of caveolae and caveosome was performed. More than 20 images (×2000 magnification) were captured from one dish and totally six dishes per each group were used and analyzed using NIH ImageJ software. The number of caveolae/caveosome was normalized with the length of cell membrane which determined between first and last caveolae/caveosome as shown in Fig. S1.

### 2.10 Functional nuclear isolation

Functional nuclear isolation was performed using commercially available nuclei isolation kit (Nuclei Pure Prep Isolation Kit; Sigma‐Aldrich) as reported previously [15]. Purity of nuclei was assessed by western blot using anti‐OXPHOS, anti‐GAPDH, total histone antibodies, and imaging with nuclear marker Hoechest33342 (Thermo Fisher Scientific Inc.) and mitochondrial marker (MitoTracker; Thermo Fisher Scientific Inc.). For functional experiments, isolated nuclei were used immediately.

### 2.11 Acetyl‐CoA measurement

Acetyl‐CoA measurement was performed using commercially available kit (PicoProbe Acetyl CoA Assay kit (Fluorometric); Abcam) as reported previously [15].

### 2.12 ACLY activity assay

ACLY activity was measured by malate dehydrogenase coupled method as reported previously [15].

### 2.13 Xenograft model

The adult male NOD‐SCID mice (6 weeks) were used in this experiment. The mice were housed in standard cages in a temperature and humidity‐controlled room with a 12‐h light‐dark cycle and *ad libitum* access to food and water. All experimental procedures involving mice were approved by the Institute of Laboratory Animals of Tohoku University (2018MdA‐237). Surgical procedures were performed under sterile conditions. In brief, mice were anesthetized by inhalation of isoflurane and then positioned on a rodent stereotaxic frame (SR‐6M‐HT, Narishige, Tokyo, Japan). Inhalation anesthesia was maintained during operation. After disinfecting the head of the mouse with iodine and incising, a hole was made in the skull using dental drill (1 mm forward, 2 mm right lateral from the bregma). Subsequently, 100 000 glioma cells in 2 μL of cold PBS were injected into brain (2.5 mm down from the dura) using a Hamilton syringe with a 27 G needle. Before injection, the needle was left in place for 3 min and after injection, 5 min before withdrawal. Before suturing, dental resin was spread over hole to prevent leaking of cells. Following transplantation, animals were monitored every day for up to 21 days. For FABP7 induction in transplanted cells, mice were treated with 2 mg·mL^−1^ doxycycline in 5% sucrose drinking water from day *in vitro* (DIV) 4 and changed every 2 days as reported previously [23]. We checked tumor growth at DIV4, DIV7, and DIV14 by *in vivo* luciferase imaging using IVIS system (*in vivo* imaging system). Transplanted glioma was harvested from mouse brain at DIV 21 for biological assessments. Mice were excluded from the experiment if they developed limp palsy, lost significant weight, or were unable to drink water. First group of mice were sacrificed at DIV14 for histochemical analysis with brain samples. The other group was sacrificed at DIV21 for biological analysis with brain samples.

### 2.14 *In vivo* luciferase imaging

Mice were intraperitoneally injected with 200 mg·kg^−1^ body weight of D‐luciferin resuspended in PBS, and imaging was performed 10 min after injection using IVIS® Spectrum CT (Caliper Life Sciences, Hopkinton, MA, USA). Bioluminescent images were acquired with 30 s exposure. Data analysis for signal intensities and image comparisons were performed using Living Image software (Caliper Life Sciences). To calculate radiance for each animal, regions of interest were carefully drawn around each signal, which is expressed as radiance (photons/second/cm^2^/steradian).

### 2.15 Statistical analysis

All data represent the mean ± SEM of at least three independent experiments. Statistical comparisons of means were made by Student’s two‐tailed unpaired *t*‐test or one‐way ANOVA followed by the Tukey test for multiple comparisons. *P* values < 0.05 were considered statistically significant.

## 3 Result

### 3.1 FABP7 is highly expressed and localized in nuclei in wild‐type IDH1 glioma

**Fig. 1:**
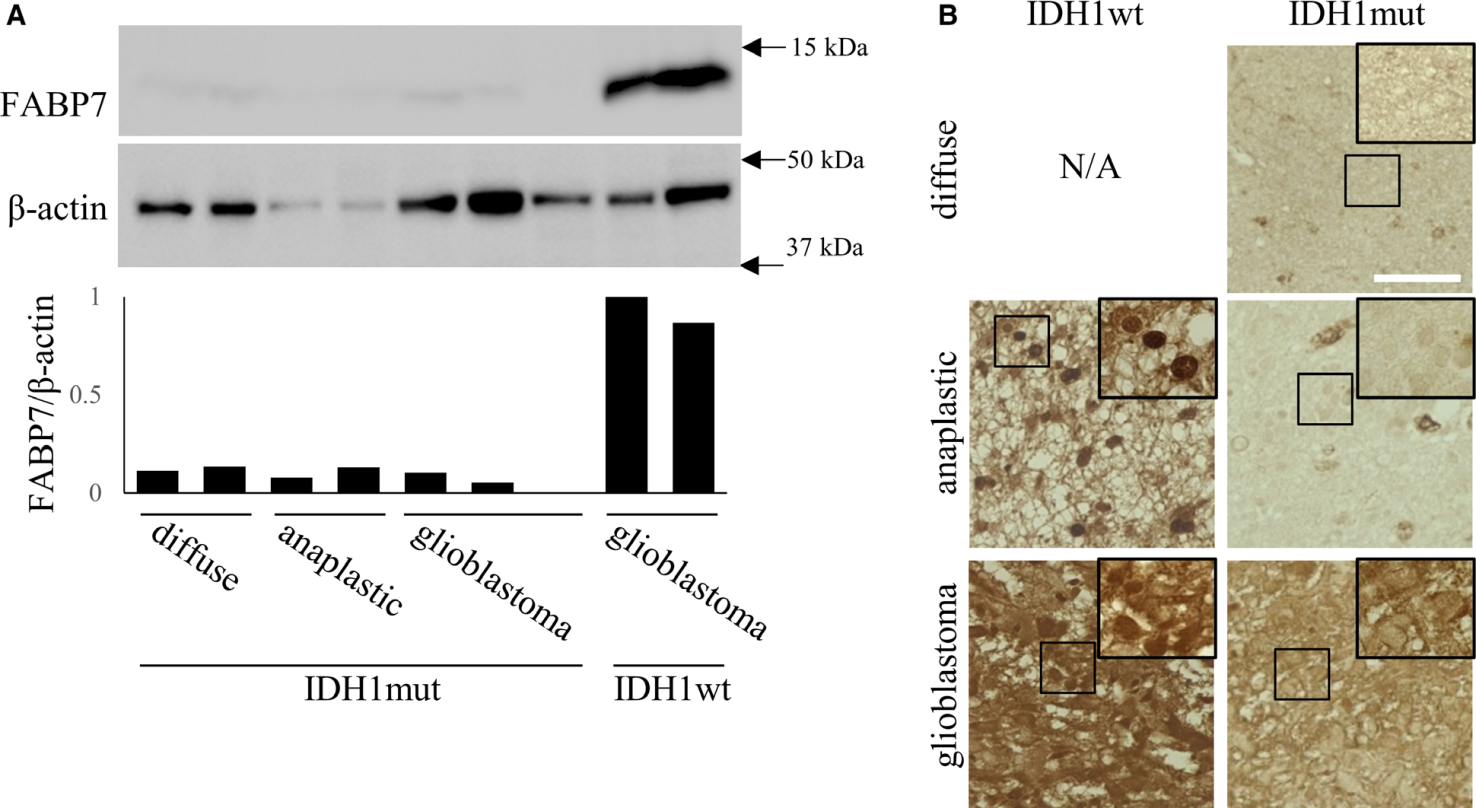
FABP7 is highly expressed and localized in nuclei in wild‐type IDH1 glioma. (A) Western blot for FABP7 and β‐actin expression in diffuse (*n* = 2), anaplastic (*n* = 2), and GB (*n* = 3) of IDH1mut patient samples and IDH1wt GB patient samples (*n* = 2). Bar graph shows band density of FABP7 normalized with β‐actin analyzed using NIH‐Image J. (B) Representative image for immunostaining of FABP7 in diffuse glioma (IDH1wt: not available, IDH1mut: *n* = 3), anaplastic glioma (IDH1wt: *n* = 3, IDH1mut: *n* = 3), and GB (IDH1wt: *n* = 3, IDH1mut: *n* = 3) of patient samples. Scale bar; 50 μm. Boxed area is enlarged on right/upper. Representative data from three experiments are shown.

We firstly evaluated the expression level of FABP7 in IDH1wt and IDH1mut glioma using Pan‐Cancer Atlas of TCGA in cBioPortal and TCGA database. Among 506 samples of diffuse glioma patients, 28 samples showed higher FABP7 expression, and notably 27 of 28 samples were IDH1wt samples (Table 1). Furthermore, when we evaluated in separated samples including astrocytoma, oligodendrocytoma, and oligodendroglioma, high frequency of FABP7‐upregulated expression was observed in IDH1wt of all types of tumors (19 of 20 in astrocytoma, 6 of 6 in oligodendrocytoma, 2 of 2 in oligodendroglioma) (Table 2). Then, we investigated FABP7 expression and localization using patient samples with diffuse astrocytoma, anaplastic astrocytoma, and GB which were diagnosed with IDH1wt or IDH1mut. Consistently with database analysis, western blot demonstrated higher expression level of FABP7 in IDH1wt GB than in IDH1mut diffuse glioma, anaplastic glioma, and GB (Fig. 1A). Notably, immunohistochemistry revealed a high expression of FABP7 in both cytoplasm and nucleus of IDH1wt GB and in nucleus of IDH1wt anaplastic astrocytoma, while IDH1mut samples expressed lower level of FABP7 without expression in nucleus (Fig. 1B). These results suggest that expression level of FABP7 is highly correlated with the presence or absence of IDH1 mutation and IDH1 mutation affects the expression and localization of FABP7.

**Table 1:** The correlation between IDH1 status and FABP7 expression level in diffuse glioma analyzed using TCGA database.

	IDH1 status	FABP7 status	% ratio (FABP7/IDH status)
Diffuse glioma (*n* = 506)	Wild‐type (*n* = 89)	Upregulated (*n* = 27)	30.34
		No‐changed (*n* = 62)	69.66
	Mutant (*n* = 417)	Upregulated (*n* = 1)	0.24
		No‐changed (*n* = 416)	99.76

**Table 2:** The correlation between IDH1 status and FABP7 expression level in astrocytoma, oligodendrocytoma, and oligodendroglioma analyzed using TCGA database.

	IDH1 status	FABP7 status	% ratio (FABP7/IDH status)
Astrocytoma (*n* = 192)	Wild‐type (*n* = 56)	Upregulated (*n* = 19)	33.93
		No‐changed (*n* = 37)	66.07
	Mutant (*n* = 136)	Upregulated (*n* = 1)	0.734
		No‐changed (*n* = 135)	99.26
Oligodendrocytoma (*n* = 131)	Wild‐type (*n* = 16)	Upregulated (*n* = 6)	37.5
		No‐changed (*n* = 10)	62.5
	Mutant (*n* = 115)	Upregulated (*n* = 0)	0
		No‐changed (*n* = 115)	100
Oligodendroglioma (*n* = 181)	Wild‐type (*n* = 17)	Upregulated (*n* = 2)	11.76
		No‐changed (*n* = 15)	88.24
	Mutant (*n* = 164)	Upregulated (*n* = 0)	0
		No‐changed (*n* = 164)	100

### 3.2 FABP7 is involved in cell proliferation rate through the regulation of caveolin‐1 expression and caveolae/caveosome function

**Fig. 2:**
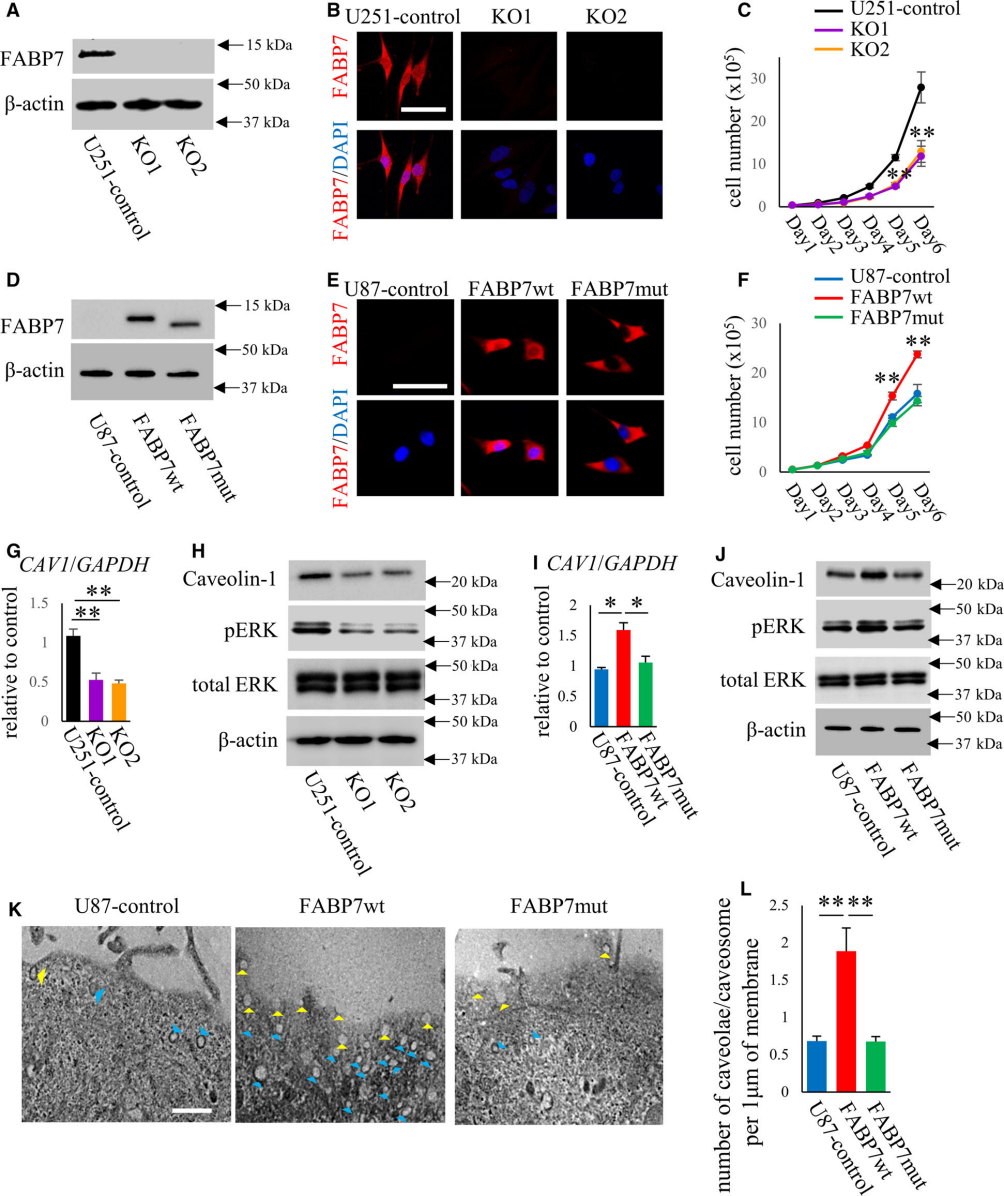
FABP7 is involved in cell proliferation rate through the regulation of caveolin‐1 expression and caveolae/caveosome function. (A) Western blot for FABP7 in U251 control and Crispr/Cas9‐edited FABP7‐KO cells. (B) Immunofluorescence staining of FABP7 (red) and DAPI (blue) in U251 control and FABP7‐KO cells. Scale bar: 50 μm (C) Proliferation rate of U251 control and FABP7‐KO cells measured successively for 6 days. One‐way ANOVA followed by the Tukey test was used for multiple comparisons. Data shown are the means ± SEM (*n* = 3) **P < 0.01 versus U251‐control (D) western blot for FABP7 in U87 cells with doxycycline‐induced control, FABP7wt, and FABP7mut. (E) Immunofluorescence staining of FABP7 (red) and DAPI (blue) in U87 cells with doxycycline‐induced control, FABP7wt, and FABP7mut. Scale bar: 50 μm (F) Proliferation rate of U87 cells with doxycycline‐induced control, FABP7wt and FABP7mut measured successively for 6 days. One‐way ANOVA followed by the Tukey test was used for multiple comparisons. Data shown are the means ± SEM (*n* = 3) **P < 0.01 versus U87‐control (G) qPCR for caveolin‐1 expression in U251 control and FABP7‐KO cells. GAPDH was used as endogenous control. One‐way ANOVA followed by the Tukey test was used for multiple comparisons. Data shown are the means ± SEM (*n* = 3) **P < 0.01 (H) western blot for caveolin‐1, Perk, and total ERK in U251 control and FABP7‐KO cells. (I) qPCR for caveolin‐1 expression in U87 cells with doxycycline‐induced control, FABP7wt, and FABP7mut. GAPDH was used as endogenous control. One‐way ANOVA followed by the Tukey test was used for multiple comparisons. Data shown are the means ± SEM (*n* = 3) *P < 0.05 (J) western blot for caveolin‐1, pERK, and total ERK in U87 cells with doxycycline‐induced control, FABP7wt, and FABP7mut. (K, L) Electron microscopy observation of caveolae and caveosome in U87 cells with doxycycline‐induced control, FABP7wt, and FABP7mut. Caveolae (yellow arrowhead), caveosome (blue arrowhead). Scale bar: 1 μm. Bar graph showed the number of caveolae and caveosome per 1 μm of membrane length. More than 20 images (×2000 magnification) were captured from one dish and totally six dishes per each group were used and analyzed. One‐way ANOVA followed by the Tukey test was used for multiple comparisons. Data shown are the means ± SEM **P < 0.01 Representative data from three experiments are shown.

To examine the role of FABP7 in tumor biology, we constructed loss of function model using IDH1wt U251 cells, which possess endogeneous FABP7 (Fig. 2A,B). Crispr/Cas9‐edited FABP7‐KO cells demonstrated lower cell proliferation rate than control cells (Fig. 2C). Next, to examine the role of FABP7 lipid‐binding domain, which is important for nuclear translocation of FABP7 [24], we constructed doxycycline‐inducible wild‐type FABP7 (FABP7wt) and mutated FABP7 (FABP7mut) in IDH1wt U87 cells as a gain of function model. We confirmed each protein expression by western blot and immunocytochemistry (Fig. 2D,E) and found that FABP7wt localized in both nuclei and cytoplasm, but FABP7mut in only cytoplasm (Fig. 2E) as reported previously [16]. FABP7wt expression in U87 increased the proliferation rate compared with control, but FABP7mut did not affect the proliferation rate (Fig. 2F). These results suggest that FABP7 expression is strongly associated with IDH1wt glioblastoma cells.

Previously, we demonstrated that FABP7 is involved in caveolae function through the regulation of caveolin‐1 expression in astrocytes [14]. Caveolae is one subset of lipid rafts, which serve as the main source of cellular activity in response to external stimuli, suggesting a strong association between caveolae function and activity of tumor proliferation. Then, we evaluated the caveolin‐1 expression and intracellular activity in loss and gain of function models. FABP7‐KO cells showed decreased mRNA and protein expression of caveolin‐1 (Fig. 2G,H) as well as decreased phospholyration level of ERK signaling (Fig. 2H). On the other hand, FABP7wt expression in U87 cells increased caveolin‐1 expression and phospholyration level of ERK signaling, but FABP7mut expression did not (Fig. 2I,J). In addition, electron microscopy analysis demonstrated that caveolae/caveosome formation was increased in FABP7wt overexpressed cells compared to control and FABP7mut overexpressed cells, but not increased in FABP7mut (Fig. 2K,L), suggesting FABP7 regulates caveolin‐1 expression as well as tumor activity through caveolae/caveosome function in glioma cells, and nuclear localization of FABP7 by appropriate function of lipid binding domain may be important in these phenominae.

### 3.3 FABP7 regulates the levels of H3K27ac on caveolin‐1 promoter

**Fig. 3:**
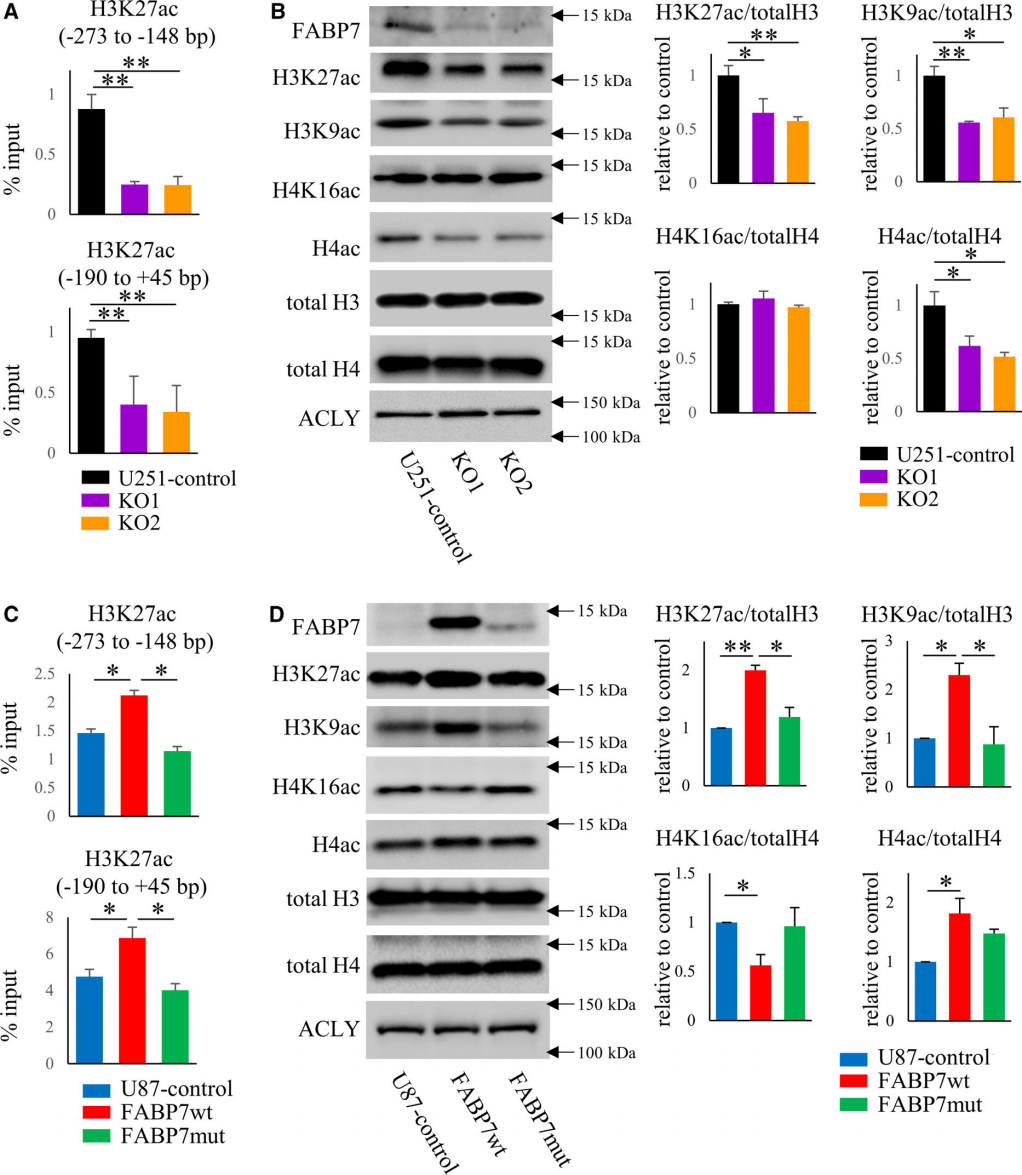
FABP7 regulates the levels of H3K27ac on caveolin‐1 promoter. (A, C) ChIP assay and subsequent qRT‐PCR with the region (−273 to −148 bp) and the region (−190 to +45 bp) on caveolin‐1 promoter for the levels of H3K27ac in (A) U251 control and FABP7‐KO cells and (C) U87 cells with doxycycline‐induced control, FABP7wt, and FABP7mut. One‐way ANOVA followed by the Tukey test was used for multiple comparisons. Data shown are the means ± SEM (*n* = 3) **P < 0.01, *P < 0.05 (B, D) western blot for FABP7, H3K27ac, H3K9ac, H4K16ac, and H4ac in isolated nuclei of (B) U251 control and FABP7‐KO cells and (D) U87 cells with doxycycline‐induced control, FABP7wt, and FABP7mut. Bar graph shows band density of H3K27ac, H3K9ac, H4K16ac, and H4ac analyzed using NIH‐ImageJ. Total H3 was used as endogenous control for H3K27ac and H3K9ac. Total H4 was used as endogenous control for H4K16ac and H4ac. One‐way ANOVA followed by the Tukey test was used for multiple comparisons. Data shown are the means ± SEM (*n* = 3) **P < 0.01, *P < 0.05 Representative data from three experiments are shown.

Recently, we demonstrated that FABP7 epigenetically regulates caveolin‐1 expression by controlling nuclear acetyl‐CoA levels through the interaction with ACLY in astrocytes [15]. To examine the role of FABP7 in GB tumors, we first examined whether FABP7 epigenetically regulates caveolin‐1 expression. Chip assay revealed that the level of H3K27ac on caveolin‐1 promoter was decreased in FABP7‐KO cells (Fig. 3A). Next, we evaluated histone acetylation using isolated nuclei from U251 control and FABP7‐KO cells with its purification confirmed (Fig. S2A,B). Interestingly, the levels of H3K27ac as well as H3K9ac and H4ac were decreased in FABP7‐KO cells compared with control (Fig. 3B). The same experiments were performed in gain of function models, and we found that FABP7wt overexpression increased the level of H3K27ac on caveolin‐1 promoter, but FABP7mut overexpression did not (Fig. 3C). Furthermore, the levels of H3K27ac as well as H3K9ac and H4ac were increased in FABP7wt compared with control, and these increases were abrogated in FABP7mut (Fig. 3D). Conversely, the level of H4K16ac was decreased in FABP7wt compared with control, but not changed in FABP7mut (Fig. 3D).

### 3.4 FABP7 regulates nuclear acetyl‐CoA levels through the interaction with ACLY

**Fig. 4:**
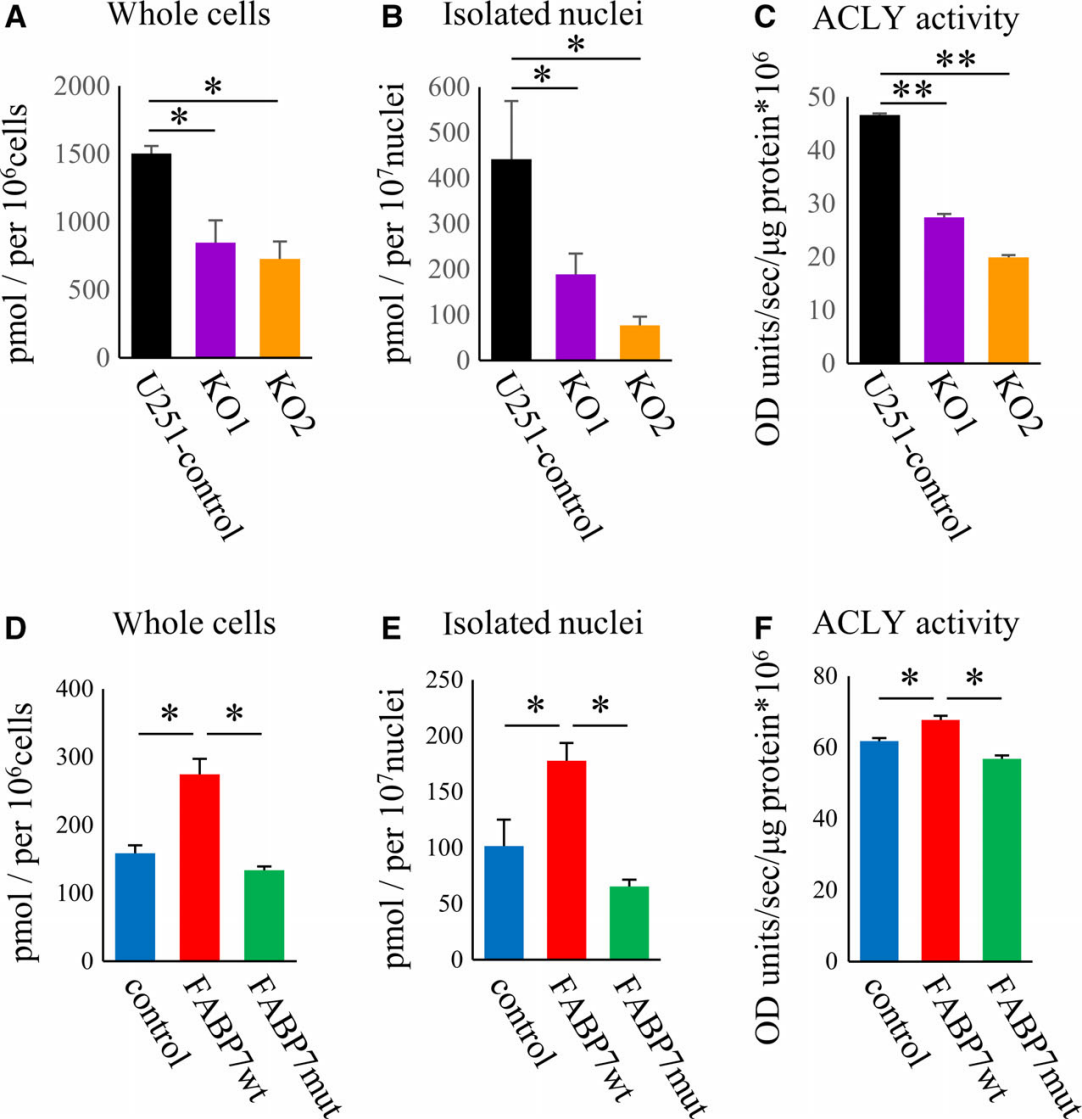
FABP7 regulates nuclear acetyl‐CoA levels through the interaction with ACLY. (A, B, D, E) Quantitative analysis of acetyl‐CoA in (A) whole cells/(B) isolated nuclei of U251 control and FABP7‐KO cells, and (D) whole cells/(E) isolated nuclei of U87 cells with doxycycline‐induced control, FABP7wt, and FABP7mut. The levels were normalized by the number of cells or nuclei, respectively. One‐way ANOVA followed by the Tukey test was used for multiple comparisons. Data shown are the means ± SEM (*n* = 3) *P < 0.05 (C, F) Measurement for ACLY activity in (C) U251 control and FABP7‐KO cells, and (F) U87 cells with doxycycline‐induced control, FABP7wt, and FABP7mut. The levels were normalized by the protein concentration. One‐way ANOVA followed by the Tukey test was used for multiple comparisons. Data shown are the means ± SEM (*n* = 3) **P < 0.01, *P < 0.05 Representative data from three experiments are shown.

We then evaluated the levels of nuclear acetyl‐CoA as well as ACLY activity. FABP7‐KO cells showed decreased level of acetyl‐CoA in nuclei and ACLY activity (Fig. 4A–C). In contrast, FABP7wt overexpression increased level of acetyl‐CoA in nuclei and ACLY activity (Fig. 4D–F), indicating nuclear localization of FABP7 in glioblastoma may be essential for the production of nuclear acetyl‐CoA through the interaction with nuclear ACLY.

### 3.5 FABP7 regulates glioma cell proliferation in xenograft model

**Fig. 5:**
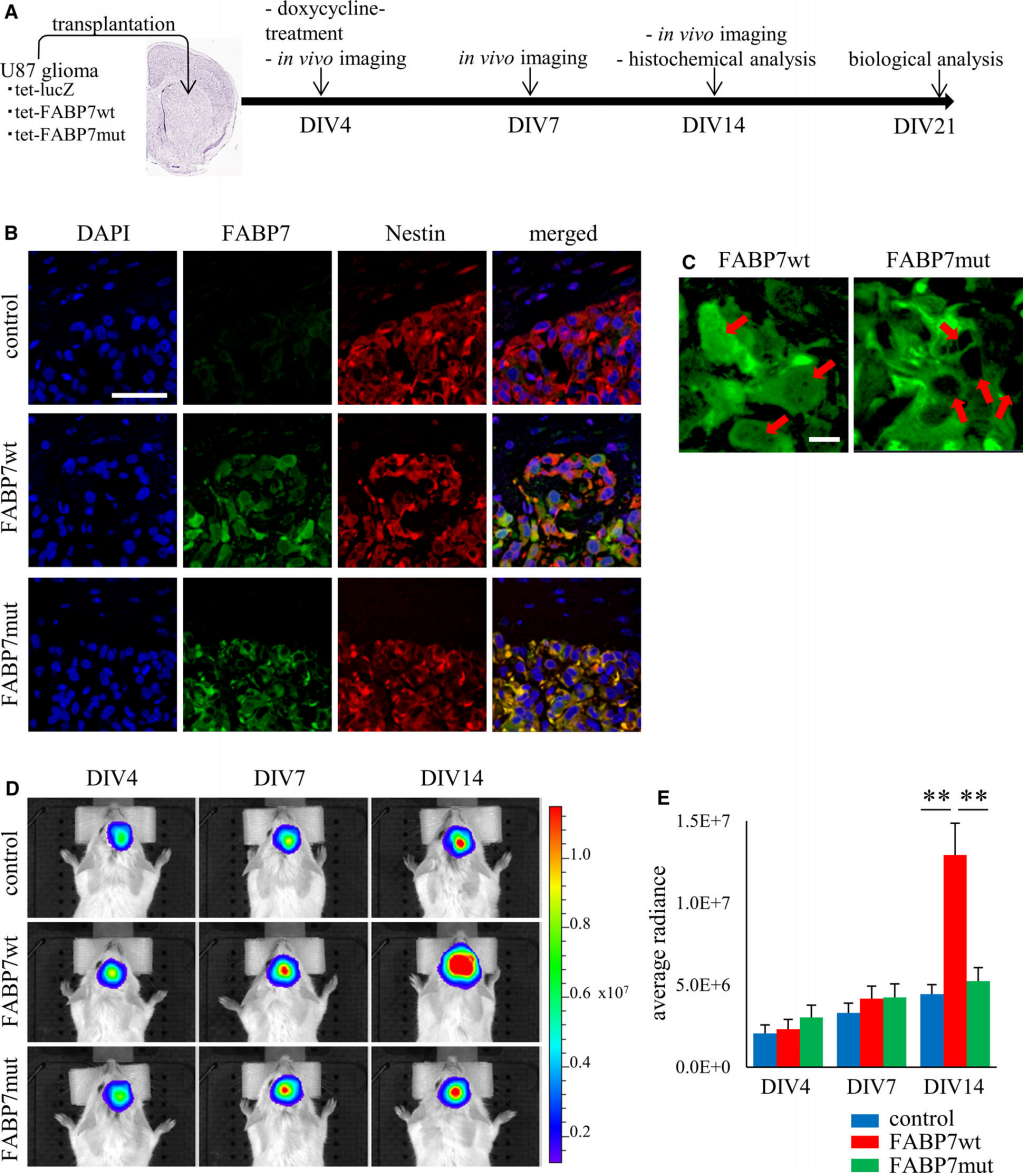
FABP7 regulates glioma cell proliferation in xenograft model. (A) Schematic representation of the time course for experiment using xenograft model. Totally, *n* = 18 mice in each group were used for experiments. Among them, *n* = 12 were used in *in vivo* imaging and biological analysis. *n* = 6 were used for histochemical analysis. (B) Immunofluorescence staining of FABP7 (green), Nestin (red), and DAPI (blue) in sectioned mouse brain transplanted with either control, FABP7wt, or FABP7mut U87 cells. Nestin was used as tumor marker. Scale bar: 50 μm (C) Representative image for localization of FABP7 (Green) in nuclei of U87 cells with doxycycline‐induced FABP7wt or FABP7mut. Scale bar: 10 μm. Red arrow indicates the position of nuclei. (D, E) *In vivo* luciferase imaging indicating tumor growth in mice transplanted with either control, FABP7wt, or FABP7mut U87 cells at indicated days. One‐way ANOVA followed by the Tukey test was used for multiple comparisons. Data shown are the means ± SEM (*n* = 6) **P < 0.01.

To examine whether FABP7 regulates tumor biology in *in vivo* system, we established the xenograft model using stable luciferase‐expressing U87 tumor. Four days after transplantation of tumors (DIV4), doxycycline treatment via drinking water was started and tumor size was evaluated by *in vivo* luciferase imaging in DIV4, DIV7, and DIV14 (Fig. 5A). The brain samples containing tumor at DIV14 was used for histological experiments and tumor biopsy samples at DIV21 in different experimental group were used for biological experiments (Fig. 5A). First, we confirmed the expression of FABP7wt or FABP7mut induced by doxycycline treatment by immunohistochemistry (Fig. 5B) and found that FABP7wt localized in nuclei and cytoplasm, while FABP7mut localized only in cytoplasm, which is consistent with *in vitro* results (Fig. 5C). Then, we traced the tumor growth in each group until DIV14. Although there is no difference in cell growth between control, FABP7wt‐ and FABP7mut‐expressing U87 cells at DIV4 and DIV7, the growth of FABP7wt‐expressing cells was significantly increased compared to control and FABP7mut‐expressing cells at DIV14 (Fig. 5D,E). Ki67 staining demonstrated that the number of positive cells was increased in FABP7wt‐expressing tumor compared to control and FABP7mut‐expressing cells (Fig. S3A). Furthermore, pERK positivity was upregulated in FABP7wt‐expressing tumor compared to control and FABP7mut‐expressing tumor (Fig. S3B), suggesting that the interaction between FABP7 and its ligands boosted the tumor proliferation through activated intracellular cell signaling in brain tumors.

### 3.6 FABP7 regulates caveolin‐1 expression, the levels of histone acetylation through the interaction with ACLY in the xenograft tumors

**Fig. 6:**
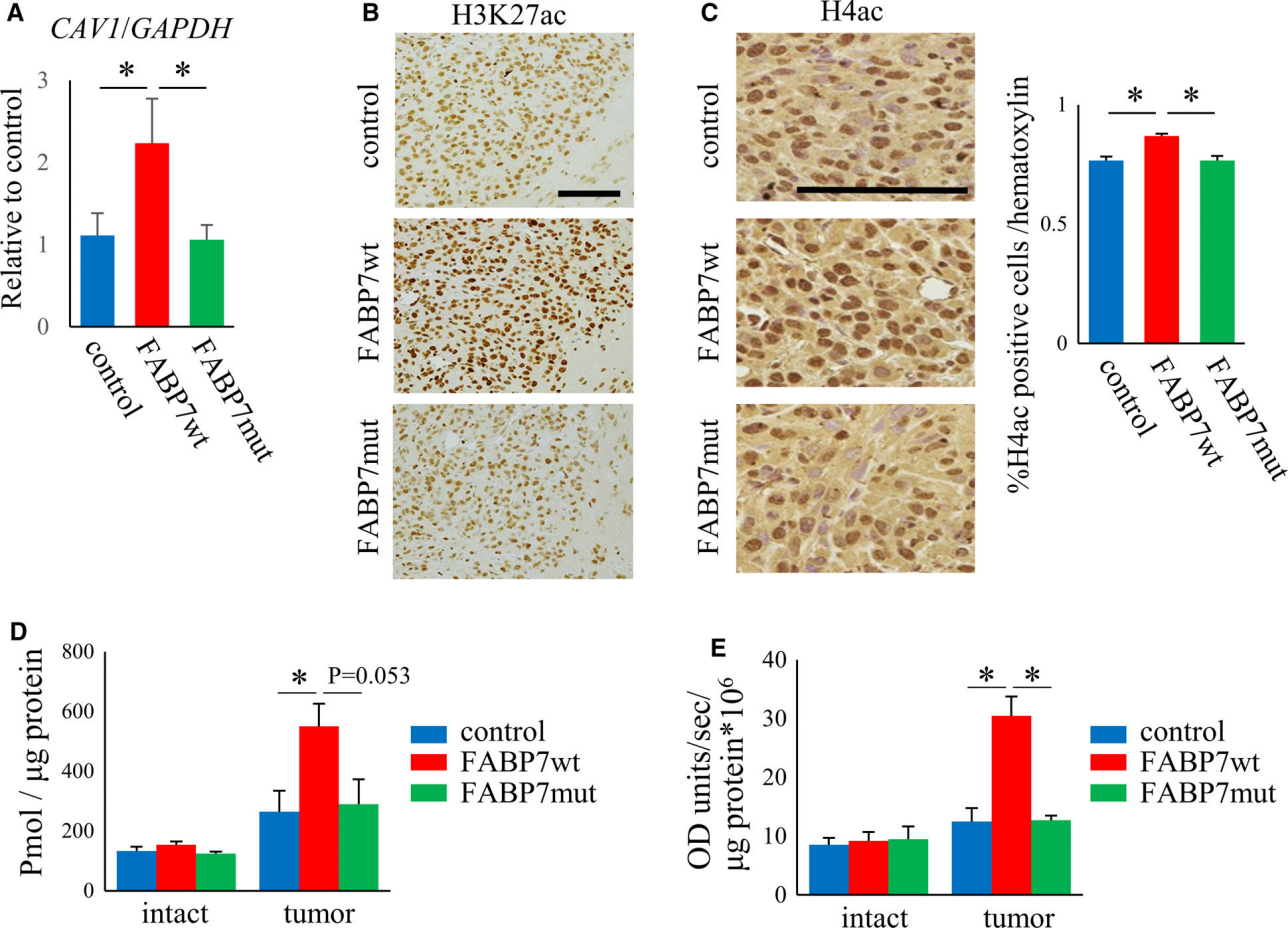
FABP7 regulates caveolin‐1 expression, the levels of histone acetylation through the interaction with ACLY in the xenograft tumors. (A) qPCR for caveolin‐1 expression in biopsy samples (DIV14) of either control, FABP7wt, or FABP7mut U87 cells. GAPDH was used as endogenous control. One‐way ANOVA followed by the Tukey test was used for multiple comparisons. Data shown are the means ± SEM (*n* = 6) *P < 0.05 (B) Representative image for immunostaining of H3K27ac in biopsy samples (DIV14) of either control, FABP7wt, or FABP7mut U87 cells. Scale: 100 μm. (C) Representative image for immunostaining of H4ac in biopsy samples (DIV14) of either control, FABP7wt, or FABP7mut U87 cells. Scale: 100 μm. Bar graph shows the analyzed data for percentage of H4ac‐positive cells per hematoxylin. One‐way ANOVA followed by the Tukey test was used for multiple comparisons. Data shown are the means ± SEM (*n* = 6) *P < 0.05 (D) Quantitative analysis of acetyl‐CoA in biopsy samples (DIV21) of either control, FABP7wt, or FABP7mut U87 cells. Intact samples were obtained from contralateral region from implanted tumor. One‐way ANOVA followed by the Tukey test was used for multiple comparisons. Data shown are the means ± SEM (*n* = 6) *P < 0.05 (E) Measurement for ACLY activity in biopsy samples (DIV21) of either control, FABP7wt, or FABP7mut U87 cells. One‐way ANOVA followed by the Tukey test was used for multiple comparisons. Data shown are the means ± SEM (*n* = 6) *P < 0.05.

At DIV21, xenograft tumors were isolated from brain. qPCR demonstrated that caveolin‐1 expression was significantly increased in FABP7wt‐expressing tumors compared to control and FABP7mut (Fig. 6A). Immunohistological analysis against acetylated H3K27 showed significant higher density in FABP7wt‐expressing tumor compared to control and FABP7mut although all tumor cells showed positivity (Fig. 6B). Staining against histone H4 acetylation was significantly higher positivity in FABP7wt‐expressing tumor compared to control and FABP7mut (Fig. 6C). Consistent with *in vitro* results, the levels of acetyl‐CoA and ACLY activity were upregulated in FABP7wt compared to control, but not changed in FABP7mut (Fig. 6D,E), suggesting that the interaction between FABP7 and its ligands regulates caveolin‐1 expression through histone modification in brain tumors.

### 3.7 Caveolin‐1 expression, the levels of histone acetylation, and the levels of acetyl‐CoA were upregulated in IDH1wt patient GB compared to IDH1mut

**Fig. 7:**
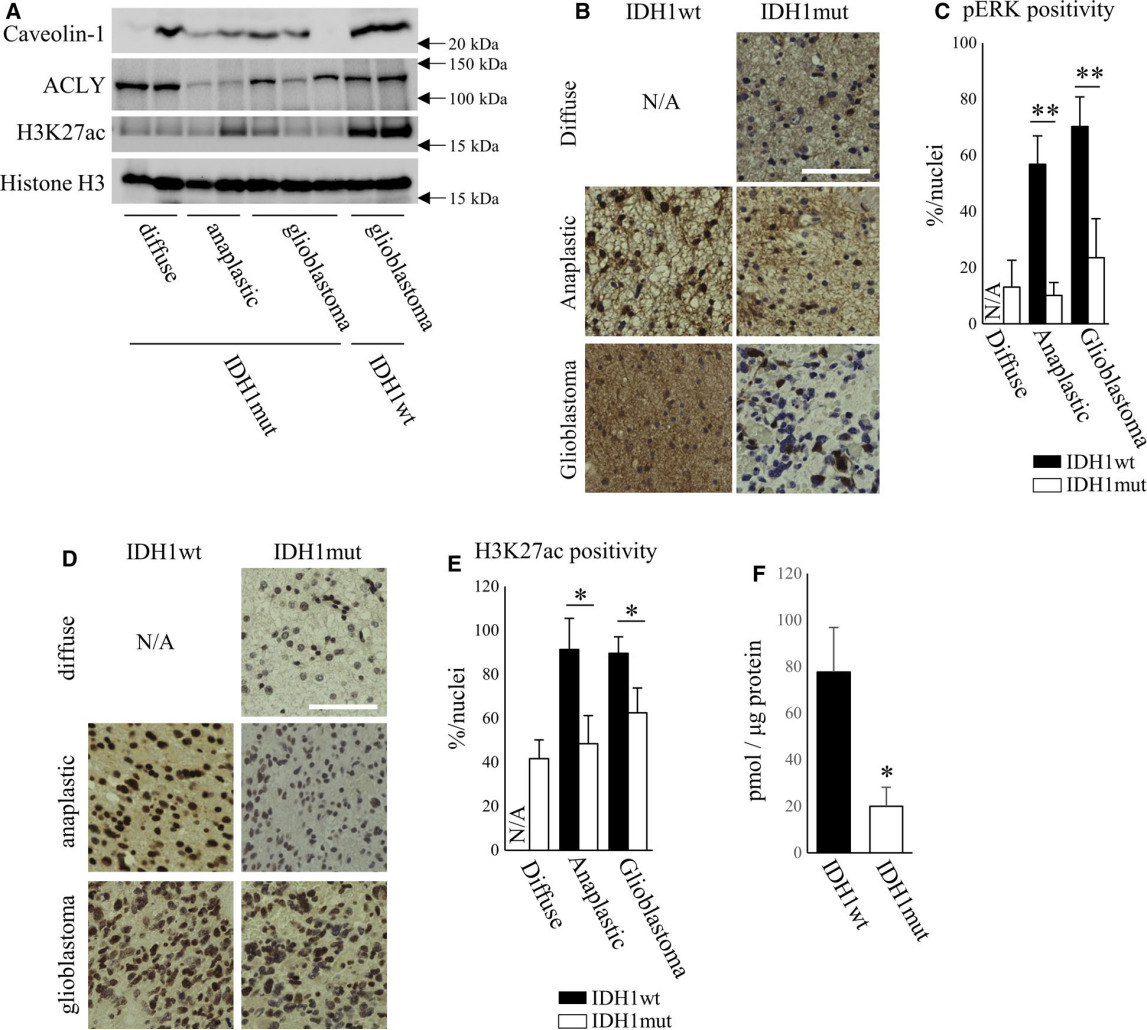
Caveolin‐1 expression, the levels of histone acetylation, and the levels of acetyl‐CoA were upregulated in IDH1wt patient GB compared to IDH1mut. (A) Western blot for caveolin‐1, ACLY, H3K27ac, and β‐actin expression in diffuse glioma (*n* = 2), anaplastic glioma (*n* = 2), and GB (*n* = 3) of IDH1mut patient samples and IDH1wt GB (*n* = 2) patient samples. (B, C) Representative image for immunostaining of pERK in diffuse glioma (IDH1wt: not available, IDH1mut: *n* = 3), anaplastic glioma (IDH1wt: *n* = 3, IDH1mut: *n* = 3), and GB (IDH1wt: *n* = 3, IDH1mut: *n* = 3) of patient samples. Scale bar; 50 μm. Bar graph shows the analyzed data for percentage of pERK‐positive cells per hematoxylin. One‐way ANOVA followed by the Tukey test was used for multiple comparisons. Data shown are the means ± SEM **P < 0.01 (D, E) Representative image for immunostaining of H3K27ac in diffuse glioma (IDH1wt: not available, IDH1mut: *n* = 3), anaplastic glioma (IDH1wt: *n* = 3, IDH1mut: *n* = 3), and GB (IDH1wt: *n* = 3, IDH1mut: *n* = 3) of patient samples. Scale bar: 50 μm. Bar graph shows the analyzed data for percentage of H3K27ac positive cells per hematoxylin. One‐way ANOVA followed by the Tukey test was used for multiple comparisons. Data shown are the means ± SEM *P < 0.05 (F) Quantitative analysis of acetyl‐CoA in patients sample of IDH1wt or IDH1mut. Student’s two‐tailed unpaired *t*‐test was used for comparison. Data shown are the means ± SEM (*n* = 6) *P < 0.05 Representative data from three experiments are shown.

Finally, we confirmed our experimental results using patient samples. We found that IDH1wt GB showed higher level of caveolin‐1 expression and activated pERK compared to IDH1mut GB (Fig. 7A–C). Consistently, the higher level of H3K27ac was observed in IDH1wt GB compared to IDH1mut (Fig. 7A,D,E). Although a significant difference was not observed in ACLY expression between IDH1wt and IDH1mut GB (Fig. 7A), the level of acetyl‐CoA was significantly higher in IDH1wt GB (Fig. 7F).

## 4 Discussion

**Fig. 8:**
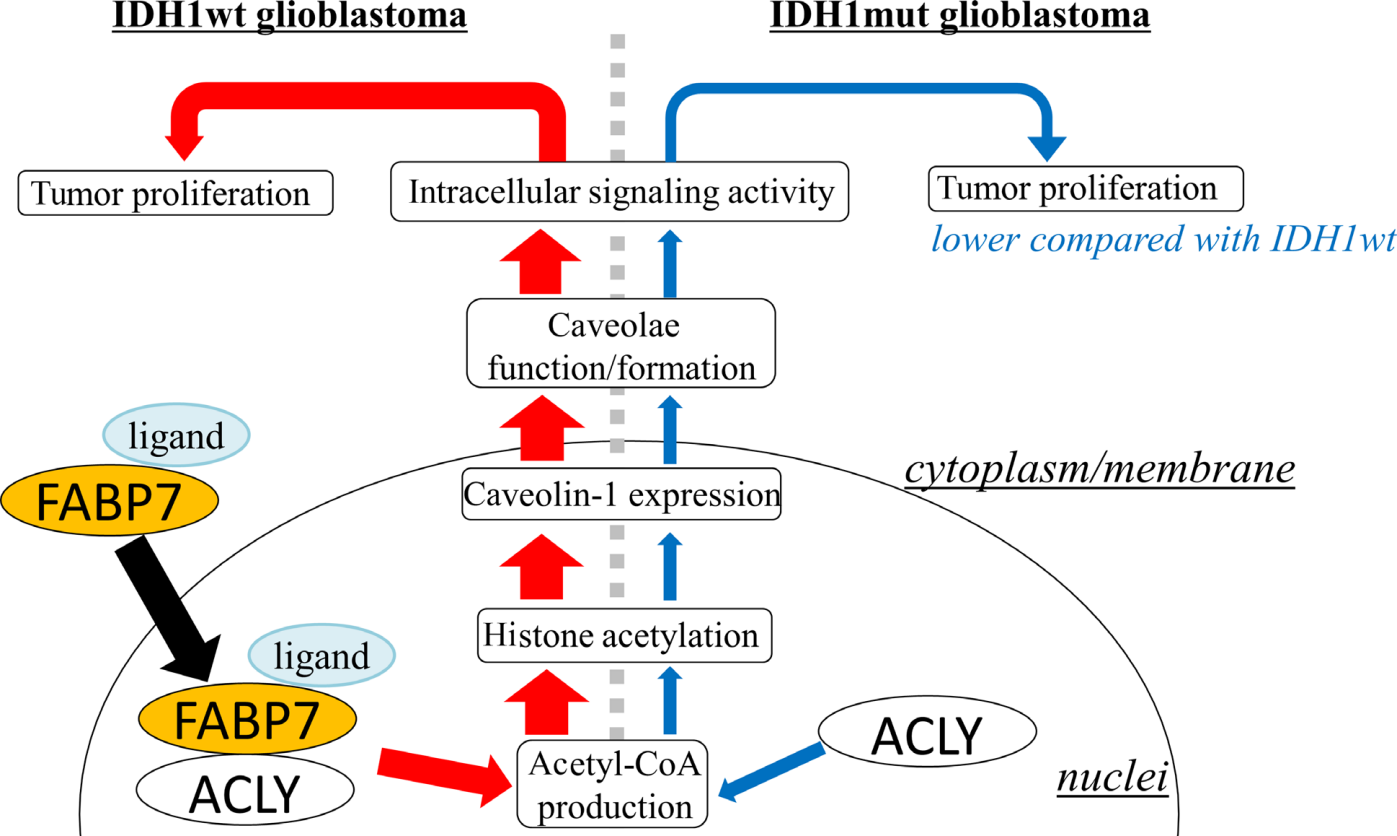
Schematic representation of the role of FABP7 in IDH1wt glioblastoma. In IDH1wt GB, ligand bound FABP7 translocates to the nucleus and increases nuclear acetyl‐CoA levels via interaction with ACLY. This leads to increase in histone acetylation of caveolin‐1 promoter and consequently caveolin‐1 expression resulting in increased caveolae/caveosome function/formation, and intracellular signaling activity driving tumor proliferation.

IDH1, a key enzyme that functions at the crossroads of cellular metabolism and epigenetic regulation, primarily catalyzes the oxidative decarboxylation of isocitrate to generate α‐ketoglutarate (αKG). At the onset of a missense mutation in the IDH1 Arg132 codon causing the single amino acid substitution to histidine (IDH1 R132H), there is catalysis of the neomorphic production of an oncometabolite D‐2 hydroxyglutarate (D2HG). D2HG, a structurally similar metabolite to αKG, has been demonstrated to function as a competitive inhibitor αKG‐dependent dioxygenase that are involved in the regulation of epigenetics and differentiation [25]. Experimental as well as clinical evidence suggests that D2HG‐induced dysregulation of histone and DNA methylation impedes normal cellular differentiation including CNS tissues [26, 27, 28], giving the high possibility of IDH1mut being a trigger for the generation of tumors. However, it is important to note that the survival span of IDH1wt patient is shorter than IDH1mut patients because IDH1mut is observed in the vast majority of lower grade glioma, while IDH1wt is often observed in GB. Thus, elucidation of IDH1wt tumor biology is critically essential. Herein, we identified high expression of FABP7 in IDH1wt anaplastic glioma and GB compared to IDH1mut glioma and found that FABP7 is highly localized in nucleus in IDH1wt anaplastic glioma and GB. Furthermore, we examined the role of FABP7 in tumor proliferation using *in vitro* model and found that FABP7overexpression activated caveolae function and formation through the epigenetic regulation of caveolin‐1 via controlling nuclear acetyl‐CoA levels. Correlatedly, IDH1wt GB showed high expression of caveolin‐1, high acetylation levels of H3K27, and high production of acetyl‐CoA. These results indicate the important roles of FABP7 in tumor biology of IDH1wt GB as shown in the schematic representation (Fig. 8).

FABP7 has three motifs that form β‐strands along with functional domains and these motifs make available the sites for binding with FABP7 ligands. Ligand binding changes the three‐dimensional structure of FABP7, exposing the nuclear localization signal as it is not visible in the primary sequence [24]. It has been reported that docosahexaenoic acid (DHA), which is preferentially bound by FABP7, induced nuclear translocation of FABP7, followed by higher rate of migration [16]. In addition, although wild‐type FABP7 is shown to facilitate melanoma proliferation and glioma metastasis [16, 29], the mutation in lipid‐binding domain of FABP7, which makes nuclear localization impossible, does not change these effect, suggesting that the interaction between FABP7 and its ligands is critically essential for nuclear localization of FABP7 and activity in tumor biology. Interestingly, it has been reported that IDH1mut induced altered lipid metabolism. Doxycycline–driven IDH1mut overexpression in U251 impaired the PUFAs contents in endoplasmic reticulum and golgi [30] and cholesterol homeostasis [31], suggesting altered intracellular lipid contents may affect the localization and expression of FABP7. Furthermore, there may be a difference in the therapeutic target is different between IDH1wt and IDH1mut glioma considering the different lipid metabolite.

Given that lipids, especially FAs, are relatively insoluble and pose detrimental cytotoxic effects in free form, cells employ the use of lipid chaperones, FABPs, for intracellular trafficking of FAs. FABP7 is shown to have high affinity for n‐3 PUFAs, EPA (Kd 48 nm), and DHA (Kd 53 nm), monounsaturated n‐9 Oleic acid (Kd 47 nm) [32, 33], and these FAs have been reported to have a strong association with tumor biology [34, 35, 36]. Furthermore, as ligand binding induces nuclear translocation of FABP7, lipid‐binding domain of FABP7 is thus essential for the role of FABP7 in the cell. Indeed, mutated FABP7 is restricted only in the cytoplasm, altering the rate of glioma proliferation [16]. In this study, we clearly demonstrated that FABP7mut disrupted the phenotypes shown in FABP7wt overexpressed cells. Notably, in xenograft model, FABP7wt overexpressed U87 showed higher proliferation rate compared to control, but FABP7mut overexpressed cells did not change the proliferation rate, indicating the interaction between FABP7 and its ligands recruited from neovascularization is associated with glioma proliferation. Although the ligands of FABP7 for tumor proliferation have not been specified, further investigation will determine this.

In this study, we have demonstrated that FABP7wt overexpression in U87 glioma cells increased caveolin‐1 expression and caveolae formation, triggering activated intracellular signaling including ERK. So far, the functional significance of lipid rafts or their related proteins in the regulation of tumor cell biology have been reported in several studies. For example, in U251 GB cells, it has been demonstrated that connexin43 interacts with caveolin‐1 in lipid rafts leading to promoting of invasion [37]. Moreover, glioma chemotaxis in response to stimuli is shown to require the association of transient receptor potential canonical channels with lipid rafts [38]. Furthermore, in melanoma cells, increased recruitment of adhesion receptors to the lipid raft after HLA‐DA stimulation and activation of intracellular signaling lead to an increase in migration and invasion [39], while treatment of cells with methyl‐β‐cyclodextrin, altering the lipid raft function, suppresses migration of melanoma cell lines [40]. Considering these data suggesting a tight association between lipid raft function and tumor proliferation, FABP7 is strongly involved in tumor activation through controlling caveolae formation.

Nuclear acetyl‐CoA, generated by the citrate‐ACLY pathway, is an essential molecule for signaling and epigenetics, acting as an acetyl donor for histone lysine acetylation [41, 42]. Previously, we demonstrated that FABP7 interacts with ACLY and regulates nuclear acetyl‐CoA levels in astrocytes [15]. In this study, we have explored the role of FABP7 in the production of nuclear acetyl‐CoA in pathophysiological condition. Although FABP7wt overexpression did not change ACLY expression, we found that FABP7wt overexpression increased ACLY activity as well as nuclear acetyl‐CoA levels and the levels of histone acetylation. Furthermore, the acetylation level of H3K27 on caveolin‐1 promoter was increased by FABP7wt overexpression, suggesting FABP7 regulates caveolae formation through the interaction with ACLY, leading to activated tumor proliferation.

## 5 Conclusion

In this study, we newly demonstrated that FABP7 is highly expressed and localized in nuclei in IDH1wt glioma. Using IDH1wt U87 cells, we demonstrated that FABP7wt overexpression increased cell proliferation rate as well as caveolin‐1 expression and caveolae formation. In addition, FABP7wt overexpression increased the levels of H3K27ac on caveolin‐1 promoter through controlling the nuclear acetyl‐CoA level via the interaction with ACLY. Consistent results were obtained using xenograft model transplanted with U87 cells containing FABP7 overexpression. Furthermore, IDH1wt patient GBM showed upregulation in caveolin‐1 expression, the levels of histone acetylation and the levels of acetyl‐CoA compared with IDH1mut patient GBM. Taken together, these data suggest that nuclear FABP7 is involved in cell proliferation of GBM through caveolae function/formation regulated via epigenetic regulation of caveolin‐1, and this mechanism is critically important for the IDH1wt tumor biology.

## Conflict of interest

The authors declare no conflict of interest.

## Author contributions

YK and YO conceived and designed the study and wrote the manuscript with comments from all authors. YK, BU, MK, RZ, SS, HM, SK, TW, and SY performed experiments. MK and TT prepared patient glioma samples and provided and technical assistance.

### Peer Review

The peer review history for this article is available at https://publons.com/publon/10.1002/1878‐0261.13130.

## Supporting information


**Fig. S1**. Proliferation assay using cell count reagent and the method for counting of caveolae/caveosome.
**Fig. S2**. The purity of functional nuclear isolation.
**Fig. S3**. FABP7 overexpressed U87 cells in xenograft model showed increased Ki67 and pERK positivity.

**Table S1**. The list of antibodies used in this study.

## Data Availability

The datasets and materials in this study are available on the reasonable request from corresponding authors.
