# Supplementary material for: Nuclear FABP7 regulates cell proliferation of wild‐type IDH1 glioma through caveolae formation
Source: Mol Oncol. 2021 Nov 9;16(1):289–306. doi: 10.1002/1878-0261.13130 (PMC8732344; doi:10.1002/1878-0261.13130)
Supplement: Supplementary file 1 — Fig. S1. Proliferation assay using cell count reagent and the method for counting of caveolae/caveosome. Fig. S2. The purity of functional nuclear isolation. Fig. S3. FABP7 overexpressed U87 cells in xenograft model showed increased Ki67 and pERK positivity. [file MOL2-16-289-s001.pdf]

Supplemental Figure 1

A

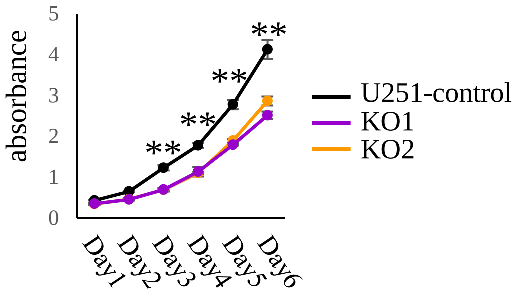

B

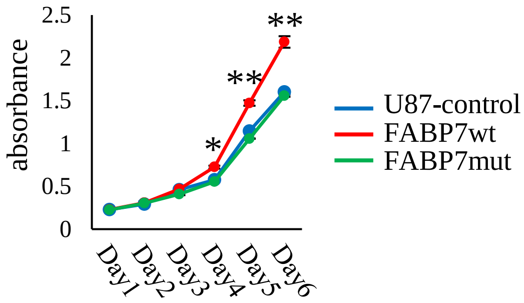

C

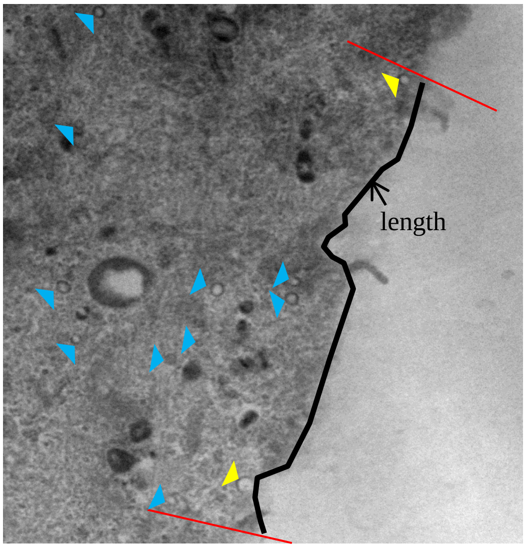

Figure S1. Proliferation assay using cell count reagent and the method for counting of of caveolae/caveosome (A, B) Proliferation rate measured successively for six days using cell count reagent SF. (A) U251 control cells, FABP7-KO cells, (B) U87 cells with doxycycline-induced control, FABP7wt and FABP7mut. Data shown are the means ± s.e.m. (n=3) $*p<0.05$, $**p<0.01$ versus control. Representative data from 3 experiments are shown. (C) Electron microgram showing the method for counting of caveolae/caveosome. The red lines indicate the edge of caveolae/caveosome and the length between lines is calculated with NIH image J software. Within red line, observed caveolae and caveosome are counted and normalized with length.

Supplemental Figure 2

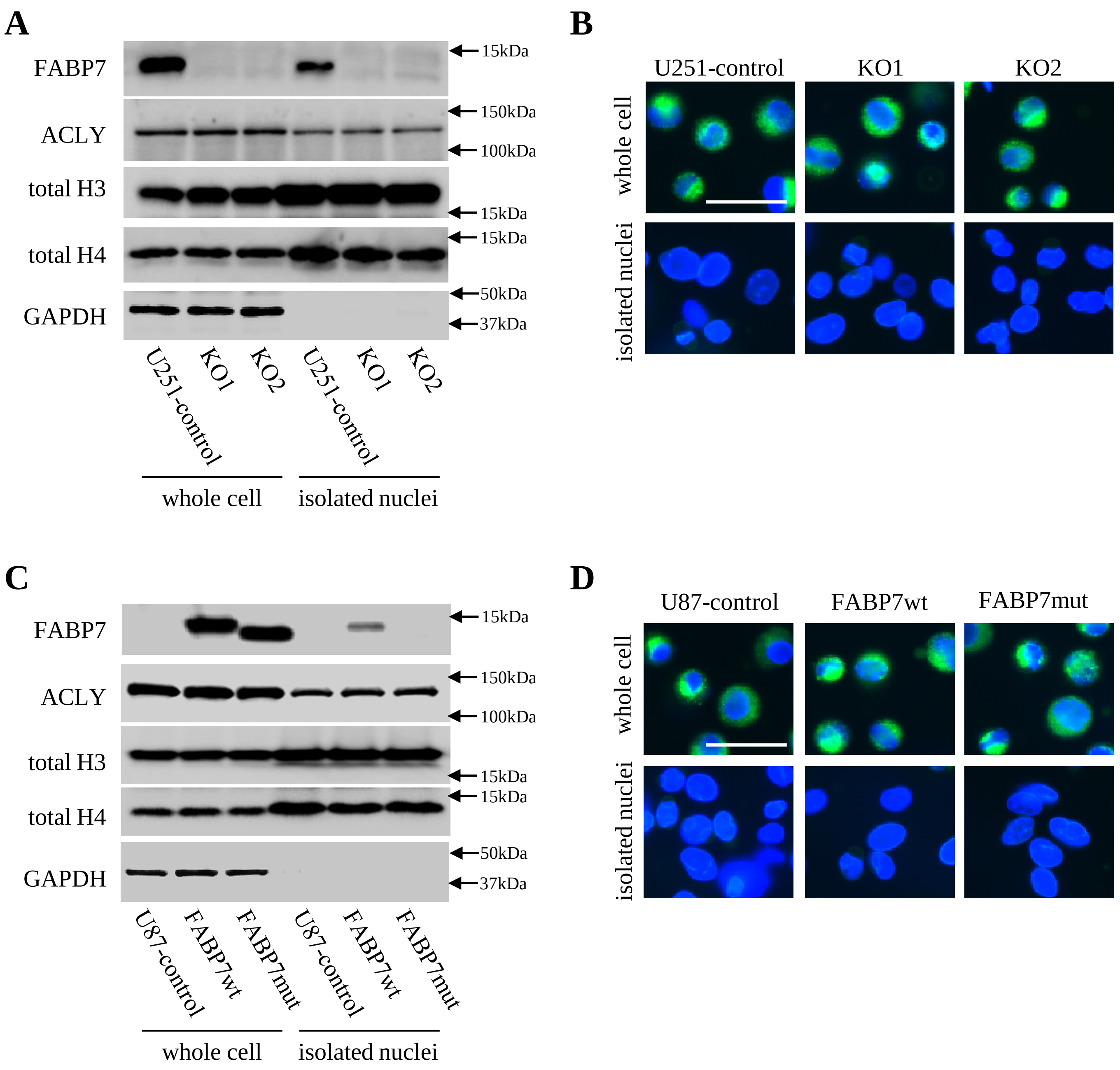

Figure S2. The purity of functional nuclear isolation (A, B) Western blot for FABP7, ACLY, total H3, total H4, and GAPDH expression in whole cell lysate and nuclei of U251 control and FABP7-KO cells. (B) Imaging to confirm the purification of functional nuclei from U251 control and FABP7-KO cells. Hoechest33342 (blue) and MitoTracker (green) are used as the marker of nuclei and mitochondria, respectively. scale bar: 50 μm (C) Western blot for FABP7, ACLY, total H3, total H4, and GAPDH expression in whole cell lysate and nuclei of U87 cells with doxycycline-induced control, FABP7wt and FABP7mut. (D) Imaging to confirm the purification of functional nuclei from U87 cells with doxycycline-induced control, FABP7wt and FABP7mut. Hoechest33342 (blue) and MitoTracker (green) are used as the marker of nuclei and mitochondria, respectively. scale bar: 50 μm

Supplemental Figure 3

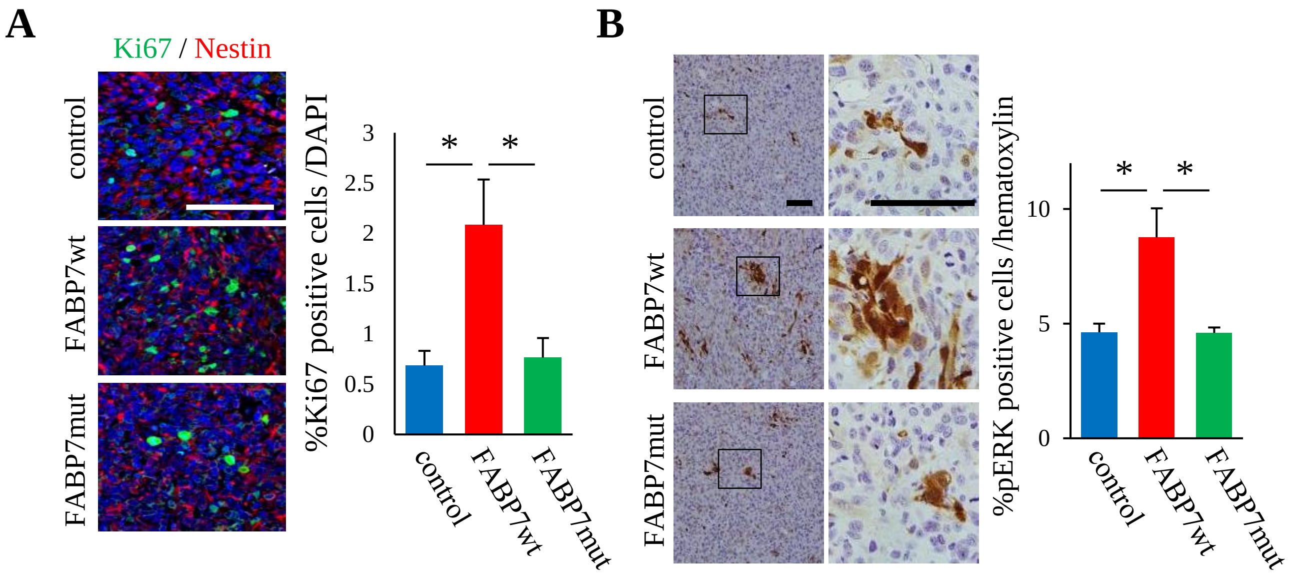

Figure S3. FABP7 overexpressed U87 cells in xenograft model showed increased Ki67 and pERK positivity. (A) Immunofluorescence staining of Ki67 (green), Nestin (red) and DAPI (blue) in control and FABP7wt/FABP7mut overexpressed U87 cells transplanted in mouse brain. Bar graph demonstrates the analyzed data for percentage of Ki67 positive cells per DAPI. Data shown are the means ± s.e.m. (n=6) *$p<0.05$ (B) Immunostaining of pERK in control and FABP7wt/FABP7mut overexpressed U87 cells transplanted in mouse brain. Bar graph demonstrates the analyzed data for percentage of pERK positive cells per hematoxylin. Data shown are the means ± s.e.m. (n=6) *$p<0.05$
