# Supplementary material for: Nuclear FABP7 regulates cell proliferation of wild‐type IDH1 glioma through caveolae formation
Source: Mol Oncol. 2021 Nov 9;16(1):289–306. doi: 10.1002/1878-0261.13130 (PMC8732344; doi:10.1002/1878-0261.13130)
Supplement: Supplementary file 2 — Table S1. The list of antibodies used in this study. [file MOL2-16-289-s002.docx]

**Supplemental Table. The list of antibodies used in this study**

| **1st antibody** | **Source** | **Identifier** |
| --- | --- | --- |
| rabbit polyclonal anti-human FABP7 | DS Pharma Biomedical Co., Ltd. (Osaka, Japan) | originally produced |
| mouse monoclonal anti-β-actin | Santa Cruz (TX, USA) | sc-47778 |
| rabbit polyclonal anti-caveolin-1 | Santa Cruz | sc-894 |
| rabbit monoclonal anti-phospho-p44/42 MAPK (Erk1/2) | CST (MA, USA) | 4370s |
| rabbit monoclonal anti- p44/42 MAPK (Erk1/2) | CST | 9102 |
| rabbit polyclonal anti-H3K27ac | Abcam (Cambridge, England) | ab4729 |
| rabbit polyclonal anti-H3K9ac | Merck Millipore (MA, USA) | 07-352 |
| rabbit monoclonal anti-H4K16ac | Abcam | ab109463 |
| rabbit monoclonal anti-H4(acetyl K5, K8, K12, K16) | Abcam | ab177790 |
| rabbit polyclonal anti-Histone H3 | Abcam | ab1791 |
| rabbit polyclonal Histone H4 | Abcam | ab10158 |
| rabbit monoclonal anti-ACLY | Abcam | 40793 |
| mouse monoclonal anti-Nestin | Abcam | ab22035 |
| rabbit polyclonal anti-GAPDH | Santa Cruz | sc-25778 |
| rabbit monoclonal anti-Ki67 | Abcam | ab16667 |
|  |  |  |
| **2nd antibody** | **Source** | **Identifier** |
| goat anti-rabbit IgG (H+L) HRP conjugated | Merck Millipore | AP307P |
| goat anti-mouse IgG HRP conjugated | Merck Millipore | AP124P |
| biotinylated rabbit anti-goat IgG | Vector Laboratory (CA, USA) | BA-5000 |
| goat anti-rabbit IgG (H+L) Alexa Fluor 488 conjugate | Thermo Fisher Scientific (MA, USA) | A27034 |
| goat anti-rabbit IgG (H+L) Alexa Fluor 594 conjugate | Thermo Fisher Scientific | A-11012 |
| goat anti-mouse IgG (H+L) Alexa Fluor 594 conjugate | Thermo Fisher Scientific | R37121 |
